# Advances in Nanomaterial-Mediated Photothermal Cancer Therapies: Toward Clinical Applications

**DOI:** 10.3390/biomedicines9030305

**Published:** 2021-03-16

**Authors:** Hwa Seung Han, Ki Young Choi

**Affiliations:** 1 Natural Product Informatics Research Center, Korea Institute of Science and Technology (KIST), Gangneung 25451, Korea; hanhwaseung@kist.re.kr; 2 Division of Bio-Medical Science and Technology, KIST School, University of Science and Technology (UST), Seoul 02792, Korea

**Keywords:** photothermal therapy, photothermal agent, photothermal effect, clinical application, cancer therapy, nanomaterials

## Abstract

Photothermal therapy (PTT) has attracted extensive research attention as a noninvasive and selective treatment strategy for numerous cancers. PTT functions via photothermal effects induced by converting light energy into heat on near-infrared laser irradiation. Despite the great advances in PTT for cancer treatment, the photothermal therapeutics using laser devise only or non-specific small molecule PTT agents has been limited because of its low photothermal conversion efficiency, concerns about the biosafety of the photothermal agents, their low tumor accumulation, and a heat resistance of specific types of cancer. Using nanomaterials as PTT agents themselves, or for delivery of PTT agents, offers improved therapeutic outcomes with fewer side effects through enhanced photothermal conversion efficiency, accumulation of the PTT agent in the tumor tissue, and, by extension, through combination with other therapies. Herein, we review PTT’s current clinical progress and present the future outlooks for clinical applications. To better understand clinical PTT applications, we describe nanomaterial-mediated photothermal effects and their mechanism of action in the tumor microenvironment. This review also summarizes recent studies of PTT alone or in combination with other therapies. Overall, innovative and strategically designed PTT platforms are promising next-generation noninvasive cancer treatments to move closer toward clinical applications.

## 1. Introduction

Cancer is among the leading causes of morbidity and mortality worldwide, and there are about 18 million new cancer patients, and 9.5 million cancer-related deaths each year [1]. For many decades, efforts have concentrated on searching for effective therapeutics for cancer. Currently, the available cancer treatments involve surgery, chemotherapy, and radiotherapy, in which the cancer patients may suffer from severe side effects and unsatisfactory results after these treatments [2,3]. However, with the increasing understanding of oncology, many advanced cancer therapies, such as immunotherapy, gene therapy, photodynamic therapy, and photothermal therapy (PTT), have drawn attention as promising and effective anticancer treatments [4,5,6,7].

Among these different therapies, PTT is attractive therapeutics in oncology because of its noninvasive and selective therapeutic potential. In general, PTT uses photothermal effects induced by photothermal agents that convert light energy into heat, thus increasing the temperature of surrounding tissue and triggering cell death [8,9]. PTT is a promising treatment because cancer cells typically show poor endurance to heat. Further, external laser irradiation with an adjustable dosage allows the selective elimination of various types of cancers and minimizes the damage to the surrounding nonmalignant tissue.

Nevertheless, PTT using non-specific, small molecule PTT agents or an external laser device, only without PTT agents has intrinsic limitations: non-selective cell death because of accumulation of small molecule PTT agents and endogenous biological chromophores in nonmalignant tissues; the need for a high power density to achieve a therapeutic effect. However, using the high-power lasers required for efficient tumor ablation also raises concerns about the safety and the cost of the equipment required. These constraints have slowed the clinical application of PTT devices.

Extensive research has been conducted to improve the photothermal effects and tumor targetability of PTT agents and remove the biological barriers to cancer treatment. Specifically, an ideal photothermal agent would have the specific tumor-targeting ability and high photothermal conversion efficiency (PCE) without absorption disturbance from the chromophores in biological tissue. The development of photothermal agents that meet these requirements has accelerated with the advancement of PTT research [10,11]. Among them, the development of nanosized photothermal agents (NPA) that can accumulate in tumors via enhanced permeability and retention (EPR) effects and active targeting has drawn attention [12,13]. NPAs can not only achieve a higher PCE and tumor accumulation than small-molecule photothermal agents but also integrate multiple imaging modes and therapeutic functions into one platform for advanced PTT applications [14,15]. Thus, NPA-mediated PTT has been highlighted as a standalone therapy for cancer treatment given its selective cancer cell-targeting ability, i.e., avoiding damage to healthy tissue. To date, diverse NPAs have been developed for enhanced PTT-based cancer treatment, such as metal nanomaterials (platinum and gold), semiconductor nanomaterials (copper), carbon nanomaterials (carbon nanotubes and graphene), and conducting polymers (polyaniline and polypyrrole) [9,16,17].

The NPA-mediated PTT exhibited improved therapeutic effects. However, an inherent problem facing PTT remains, such as the limited light penetration depth, leading to incomplete tumor ablation. Moreover, if the target-specificity of the NPAs is low, unnecessary damage to normal tissue can occur by overheating outside of the tumor area. The overexpression of heat shock proteins is another hurdle for PTT because it can cause resistance to PTT in some types of cancers [8].

**Figure 1:**
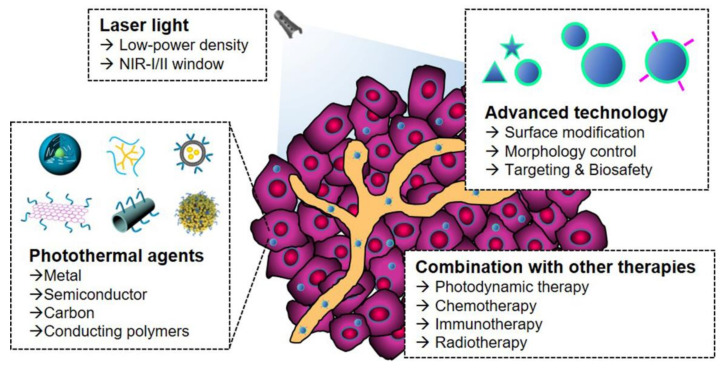
Schematic illustration for developing new PTT platforms with rational, technological innovations, and strategic improvements for clinical applications.

Many innovative strategies have been developed to improve the practicality of the PTT approach for anticancer therapy. The strategies include: (i) the identification of appropriate irradiation conditions, such as power density and time [16,17]; (ii) monitoring and optimization of the accumulation of NPA in tumor tissue [18]; (iii) improvement in PCE or targeting ability of NPA by controlling morphological properties or functionalization via surface engineering [19]; and (iv) development of NPAs with absorption in the near-infrared (NIR) II region (1000–1700 nm) [20,21] (Figure 1). PTT was also combined with different therapeutic methods, such as photodynamic therapy (PDT), immunotherapy, chemotherapy, or radiotherapy, to exploit each therapy’s advantages, and offset their limitations. Combined PTT treatment with other therapies has shown significant treatment outcomes by improving drug delivery efficiency, triggering drug release, controlling the tumor microenvironment (TME), or eliciting tumor-specific antigen release [22].

Unlike other comprehensive review articles covering a wide range of studies on NPA-mediated photothermal tumor therapies [23,24,25,26], in this review article, we highlight the clinical progress of PTT-based approaches. Furthermore, to achieve a better understanding of current clinical PTT approaches, we elaborate on the different types of NPAs and their photothermal effects and mechanisms of action in the TME, as well as concisely present examples of combined strategies involving PTT and other therapies. In addition, we discuss the importance and potential of NPA-mediated PTT and, by extension, suggest directions for the expansion of PTT to achieve substantial clinical anticancer effects. Finally, the points to be considered for future clinical applications are discussed.

## 2. Photothermal Agents and Their PTT Effects in the Tumor Microenvironment

**Figure 2:**
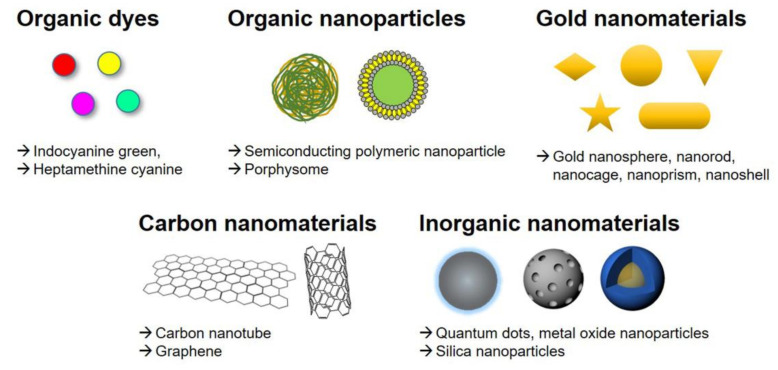
Different types of photothermal agents used for PTT.

An ideal photothermal agent should possess a high PCE at the treatment wavelength, excellent biocompatibility that includes minimal dark toxicity outside of the light-exposed tissue, and robust photostability. Generally, photothermal agents are divided into the following types: (i) organic dyes, such as indocyanine green and heptamethine cyanine; (ii) organic nanoparticles, including organic semiconducting polymeric nanoparticles and porphyrin-lipid conjugate porphysomes; (iii) gold nanomaterials; (iv) carbon-based nanomaterials such as carbon nanotubes and graphene oxide; and (v) other inorganic materials such as metal oxide nanoparticles and quantum dots (Figure 2).

Among the various photothermal agents, organic nanomaterials often have good biocompatibility and biodegradability; however, they also have inherent limitations such as low photothermal conversion efficiency, poor photothermal stability, and complicated synthesis. In contrast, inorganic nanomaterials usually show excellent NIR light absorbance; high photothermal efficiency and photostability owing to their intrinsic optical properties, such as localized surface plasmon resonance (e.g., gold nanomaterials), narrow emission spectra, and structural features that offer advantages such as ease of synthesis and surface modification with different functional groups, including carboxylic, hydroxyl, and epoxy groups (e.g., carbon-based nanomaterials). However, their non-biodegradable nature and potential long-term toxicity remain unsolved issues [27].

**Figure 3:**
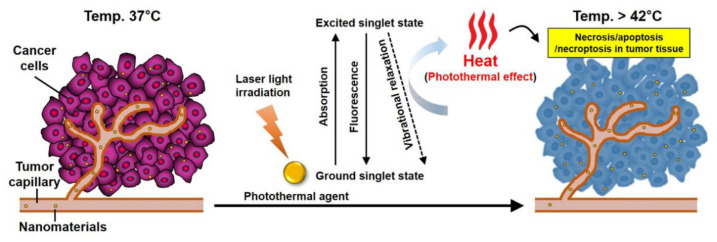
Mechanism of action for nanomaterial-mediated PTT effects in the tumor microenvironment. Nanomaterials accumulate within solid tumors that have a leaky tumor vasculature via the EPR effect. The nanomaterials have strong absorbance in the NIR window and can efficiently convert the laser energy to heat. For tumor ablation, the heat (>42 °C) generated during the excited PTT agents’ vibrational relaxation induces the photothermal effect, which results in the necrosis, apoptosis, and necroptosis of tumor tissue.

In terms of the mechanism of action, photothermal agents act as an enhancer to heat up target cells or tissues. Specifically, on irradiation by light of a specific wavelength, the agents absorb energy, and are excited from the ground singlet state to an excited singlet state. The excited PTT agent then undergoes nonradiative vibrational relaxation, returning to the ground state by a collision between the excited photothermal agents and the surrounding molecules. As a result, the increased kinetic energy heats the surrounding tumor microenvironment (Figure 3).

PTT results in several physiological and biological changes in the tumor tissue that can maximize the therapeutic effects of PTT and enhance the efficacy of secondary therapies. For example, the localized heat generation enhances the permeability of the tumor vasculature and cell membranes, which can increase the accumulation of secondary therapies, such as chemotherapy [28,29,30]. PTT also causes changes in the intracellular tumor environment, such as DNA damage and protein denaturation [31]. PTT’s cell death mechanisms may influence successful and potential combination therapies integrating PTT, chemotherapy, and immunotherapy.

Cell death via PTT is generally accompanied by two different cell signaling pathways when cells are exposed to temperatures greater than 42 °C: necrosis or apoptosis [32,33]. Moreover, recent studies have reported a new cell death mechanism in PTT, named necroptosis, which is very similar to passive, non-regulated necrotic cell death but, in contrast, is a tightly controlled cell death process [34,35,36]. When the temperature reaches 41 °C, a series of rapid changes in gene expression patterns, such as the generation of heat-shock proteins, is initiated to mitigate the effects of the initial thermal damage [37]. At 42 °C, irreversible tissue damage occurs, and heating of tissue at approximately 46 °C induces necroptosis and apoptosis [36], whereas heating of tissue above 49 °C results in cell necrosis [38]. During necrosis, heat disrupts the cell membrane, causing cytoplasmic components to leak out, and resulting in inflammation. Above 46 °C, cell death is rapidly accelerated owing to microvascular thrombosis and ischemia, finally resulting in protein denaturation and cell membrane destruction [38]. However, for apoptosis, the cell death pathway is highly regulated; inflammation is not induced, making it a suitable route to eliminate cancer cells. This distinction in the mechanism of cell-death is widely recognized in cancer treatment by PTT. Additionally, recent reports have demonstrated that necroptosis increases the sensitivity of tumor cells to antitumor treatments and results in the death of drug-resistant tumor cells via the malfunction of cell apoptosis [39]. Therefore, although there have been only a few reports on tumor cell death by necroptosis via heat treatment to date, necroptosis may be an alternative mechanism for PTT-induced cancer cell death [40,41,42].

Conventional PTT employs high-energy irradiation to cause rapid photothermal heat generation, leading to cellular necrosis. Although this strategy is sufficient for the ablation of established tumors, it can also produce undesirable effects such as unavoidable damage to the normal tissue near the tumor [43,44,45]. Recent studies have demonstrated that PTT can be modulated to trigger apoptosis, rather than necrosis by modifying experimental parameters such as laser power, exposure time, and the concentration of photothermal agents [46]. Specifically, high-energy PTT that induces necrosis can accelerate the cellular waste release and trigger damage-associated molecular patterns (DAMPs) that cause inflammation, which may induce increased secondary tumor growth. Conversely, low-energy PTT can promote cellular apoptosis, which can lead to beneficial immunogenic responses [46,47].

Interestingly, an appropriate thermal dose also generates an immune response to eliminate tumors and induces a “vaccine-like” immune response and T-cell infiltration [48]. Moreover, PTT has been used to treat local metastasis in lymph nodes and trigger the immune response for metastasis inhibition, promoting antitumor T-cell responses [49,50]. Given that the initiation of apoptosis versus necrosis with PTT can result in distinct cellular effects, researchers should carefully consider modulating the PTT parameters and utilizing PTT combined with secondary treatment strategies to maximize the therapeutic effects. Overall, modulating PTT to trigger apoptosis is a favorable option because it does not cause a pro-inflammatory response or the formation of secondary tumors.

## 3. PTT in Combination with Other Cancer Therapies

**Figure 4:**
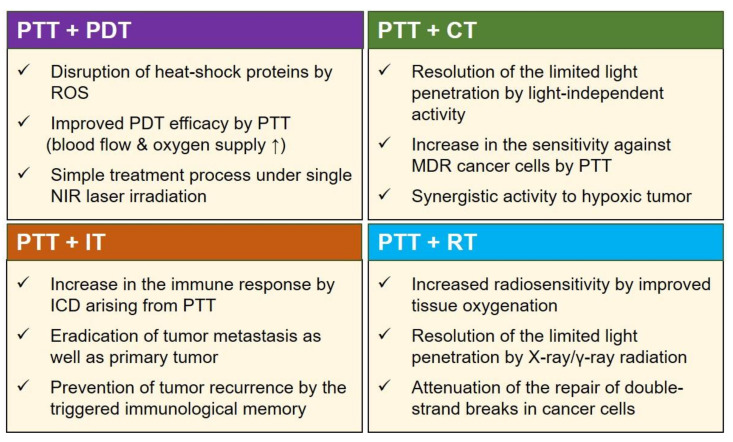
Beneficial effects of combining PTT with other cancer therapies, such as photodynamic therapy (PDT), chemotherapy (CT), immunotherapy (IT), and radiotherapy (RT). These strategies demonstrate synergy by incorporating the merits and offsetting the drawbacks of individual therapies. Abbreviations: ICD: immunogenic cell death, MDR: multi-drug resistance, NIR: near-infrared, ROS: reactive oxygen species.

The combination of PTT with other therapeutic modalities provides opportunities to exploit the advantages and offset the disadvantages of each therapeutic modality, leading to additive or even synergistic therapeutic effects (Figure 4). Further, cooperative interaction between different therapies can result in antitumor effects at low doses of photothermal agents or low laser powers, thus minimizing the potential toxicity to nonmalignant tissues. Multimodal therapies incorporating PTT could also help overcome multi-drug resistance (MDR) and hypoxia-related resistance to cancer therapy. Recent reports have demonstrated the benefits of combining PTT with other therapeutic modalities such as PDT, chemotherapy, radiotherapy, and immunotherapy [51,52,53,54]. Various nanomaterials have been used as photothermal agents in combination therapies (Table 1).

In particular, the combination of PTT and PDT can induce a potential synergistic therapeutic effect compared to PDT or PTT alone. The heating effect of PTT can enhance the delivery of the PDT agent into the intracellular environment and increase the oxygen concentration in tumor tissue by improving local blood flow, thus resulting in a higher PDT efficacy [56,74,75,76]. Additionally, reactive oxygen species (ROS) generated by PDT can obstruct heat-shock proteins, thereby hindering the protective effects of the proteins in cancer cells during PTT [77]. However, combined PTT and PDT requires sequential irradiation with lasers of different wavelengths because of the mismatched absorption spectra for the activation of the PTT agent and PDT agent, which may cause a prolonged and complicated treatment process. To overcome these drawbacks, researchers have developed simultaneous PTT and PDT using a PTT agent coupled with a PDT agent or a dual-mode PTT and PDT agent using single-laser irradiation [57,58]. Although these approaches provide a simple treatment process and improved therapeutic outcomes compared to the single-mode treatment, it requires a relatively high PTT laser power density of >1 W/cm^2^ and irradiation time of more than 5 min to trigger synergistic PTT and PDT effects. In this regard, the use of simultaneous PTT and PDT with a low NIR power density for a short irradiation time is needed to maximize the therapeutic outcome and minimize laser-induced toxicities [78].

Chemotherapy, the most common cancer treatment, still faces challenges, such as drug resistance and adverse side effects that limit the maximum administered dose. In this respect, the heating effect induced during PTT can improve the permeability of blood vessels, cell membrane, and extracellular matrix [79]. These physiological changes can boost the chemotherapeutic effect by increasing the anticancer drug content in tumor tissue or metastatic tumor tissue [80,81]. In addition, the combined use of PTT agents and chemotherapeutic agents can provide synergistic therapeutic effects; for example, the use of chemotherapy could help resolve the problems related to the limited penetration depth of light in PTT and improve the sensitivity of cancer cells to hyperthermia; further, the use of PTT could increase the therapeutic sensitivity of multidrug-resistant (MDR) cancer cells [82,83]. The combination of PTT and chemotherapy can also produce synergistic activity in hypoxic tumors owing to the increased blood flow and oxygen saturation. In a recent report, PTT combined with chemotherapy based on gold-coated nanocages containing doxorubicin showed an obvious reduction in tumor size of the pulmonary metastatic tumor models through hyperthermia-triggered drug release [60]. Besides, combined PTT and chemotherapy using polydopamine-coated spiky gold nanoparticles successfully elicited antitumor immune responses, and eliminated tumors in CT26 colon carcinoma and TC-1 lung metastasis models [51].

In addition to its combination with PDT or chemotherapy, PTT could induce a systemic anticancer immune response through localized eradication of a given tumor. Specifically, combining PTT with immunotherapy has attracted significant attention owing to its ability to elicit immune responses by immunogenic cell death (ICD), thereby augmenting the immunotherapeutic efficacy [84]. The enhanced immunotherapeutic responses induced by PTT can be explained through the following mechanisms: (i) effective destruction of the tumor tissue by local immune cells, (ii) release of tumor-specific antigen as an in situ vaccine, and (iii) activation of the immune system by pro-inflammatory cytokines [85]. Based on these mechanisms, combined PTT and immunotherapy using immunologic adjuvants can increase tumor immunogenicity and reduce immunosuppression in the TME via immune-checkpoint inhibitors, resulting in an enhancement in the tumor infiltration of cytotoxic CD8+ T-cells and effector memory T-cells. A recent report has shown that immune-checkpoint inhibition can have dramatic anticancer effects when combined with PTT and molecular adjuvants [48].

Furthermore, combining PTT with immunotherapy can modulate the immunosuppressive TME, inhibiting general obstacles such as tumor recurrence and metastasis facing single-mode PTT treatment. In one study, combined PTT treatment based on single-walled carbon nanotubes with anti-CTLA-4 effectively inhibited the growth of the secondary tumor or lung metastasis in a mouse model [68]. In another study, combined local PTT and immunotherapy using glycated chitosan induced a systemic immune response against primary tumors and metastasis in pancreatic tumor models upon direct laser irradiation [86].

Moreover, PTT can improve the therapeutic effects of RT [87,88]. Although over 50% of patients with cancer receive curative or palliative RT, in single radiation treatment, it is challenging to control the dose of ionizing radiation required to eradicate the cancerous tissue because of the radioresistance of tumor cells and the need to protect normal tissues from the radiation [89,90]. In this regard, the combination of low-energy long-wavelength NIR light and high-energy short-wavelength radiation acts in a complementary way; for example, NIR light has a limited penetration depth, but X-rays and γ-rays radiation do not have depth restrictions [91]. As mentioned earlier, studies have shown that PTT increases the temperature of tumor tissue, leading to increased tumor oxygenation, thereby sensitizing the tumor to X-ray radiation [73]. In addition, PTT can enhance the effect of RT by attenuating the repair of double-strand breaks (DSBs) in cancer cells caused by RT [92,93]. Therefore, the combination of PTT and RT can reduce the required dose of antitumor irradiation while simultaneously providing enhanced therapeutic effects [70].

## 4. Progress toward Clinical PTT Applications

**Figure 5:**
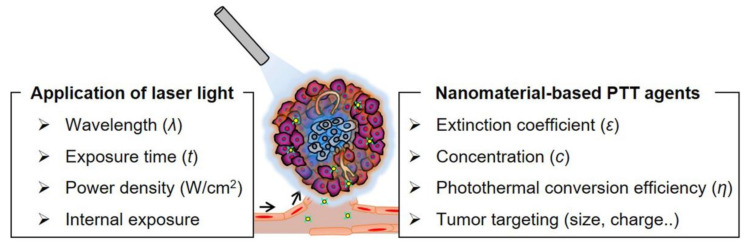
Schematic highlighting the factors crucial to achieving effective clinical PTT applications. Current research directions involve alternating the light exposure parameters to control PTT effects in tumor tissue or the use of alternative delivery methods such as intravenous or local sustained delivery in PTT.

To date, many types of nanomedicines have been approved for clinical use or are in clinical trials for cancer treatment. Despite the fascinating properties and therapeutic potential of nanomaterial-mediated PTT, clinical implementations are still lacking and face obstacles that must be overcome (Figure 5). Clinical PTT therapies are currently based on laser devices because they can achieve thermal ablation simply by exciting endogenous tissue chromophores. This strategy reduces the complexity of regulation and development costs of PTT agents. For example, endobronchial tumors can be treated with endoscopic Nd:YAG laser treatment via photocoagulation. Laser photocoagulation specifically affects the blood vessels surrounding and supplying nutrients and oxygen to the tumors and results in thermal destruction of the tumor tissue [94]. Laser treatment has also been used as a safe and effective approach for several solid tumors, such as liver and prostate tumors, under magnetic resonance imaging (MRI) guidance. For example, Nd:YAG laser treatment demonstrated therapeutic effects in 603 patients with metastatic liver from colorectal carcinoma (<5 cm in diameter) and 500 patients with hepatocellular carcinoma (<3 cm in diameter), yielding a 2% recurrence rate for tumor metastasis, and 81% ablation efficacy for primary liver cancer [95,96]. Laser treatment also resulted in minimal complications (<1% of patients) in 899 patients with malignant liver tumors [97].

In the late 2000s, the US Food and Drug Administration (FDA) approved two devices involving MRI guidance for the stereotactic laser ablation of high-grade glioma using Visualase Thermal Therapy ^®^ (150 W, 980-nm laser) and NeuroBlate ^®^ laser ablation system (12 W, 1064-nm laser).

Indocyanine green (ICG), an FDA-approved medical contrast agent for intravenous administration, has received considerable attention as a phototherapeutic agent given its photothermal effect and cytotoxic ROS generation upon NIR laser irradiation [98,99]. In particular, ICG has been used as an effective NIR-absorbing PTT agent with excellent light-to-heat conversion efficiency for cancer treatment [100,101]. In 2011, a pilot clinical study showed the feasibility of ICG for the treatment of metastatic breast cancer. A total of 10 patients with advanced-stage metastatic breast cancer received laser immunotherapy consisting of the local injection of ICG and glycated chitosan, followed by 805-nm laser irradiation at a power density of 1 W/cm^2^. NIR laser-based immunotherapy achieved an objective response rate of 62.5% and a clinical benefit response rate of 75%. There were no significant adverse events after treatment, aside from limited local thermal injury [102].

Although laser-based PTT has low regulatory hurdles and development costs, PTT-agent-enhanced thermal ablation offers significant improvements, including better selectivity to the target tissue and simple device design by using lower-power lasers. However, most studies using PTT agents, such as gold-based nanomaterials and light-absorbing nanomaterials, have only involved preclinical research, and there are only a few early-phase pilot clinical trials of PTT agent-enhanced thermal ablation, as discussed below.

Aurolase ^®^, the most prominent PTT system, is developed based on a 150-nm gold nanoshell by Nanospectra Biosciences. Aurolase ^®^ is a nanomaterial-enhanced PTT method that exploits 150-nm gold nanoshells comprised of a 120-nm silica core as the dielectric core, a 15 nm gold shell for NIR light-responsive thermal ablation, and a polyethylene glycol (PEG) layer for stable particle stability. AuroShell ^®^ absorbs energy from near-infrared light and converts it to heat, resulting in selective hyperthermic cell death with minimal damage to the adjacent nontumor tissue. For clinical trials, gold nanoshells are intravenously injected into the bloodstream and are passively accumulated in the tumor via the EPR effect. Under NIR laser irradiation, Aurolase ^®^-based therapy leads to cell death and tumor regression through PTT-induced thermal ablation. To date, there have been four recorded clinical studies based on this PTT platform on ClinicalTrials.gov. The two clinical trials involving metastatic lung tumors (NCT01679470) and head and neck tumors (NCT00848042) were completed, and two clinical studies are being carried out to investigate the therapeutic efficacy for prostate cancer (NCT02680535 and NCT04240639) (Table 2).

In the first clinical trial (NCT01679470), AuroShell ^®^ was intravenously administered to patients with primary and metastatic lung tumors, followed by NIR laser irradiation using bronchoscopy to trigger NIR-triggered thermal ablation. In the second clinical study (NCT00848042), patients with head and neck tumors received AuroShell ^®^ through intravenous administration and were given one or multiple 808-nm laser irradiation. These clinical trials were not completed, and side effects were noted in patients with refractory or recurrent head and neck cancers. However, in prostate cancer treatment, efficient focal ablation of clinically significant prostate lesions with minimal damage to healthy tissue was achieved using MRI/US-guided laser irradiation (NCT02680535). Focal laser ablation has been successfully carried out in 94% of patients without significant complications and marked changes in the International Prostate Symptom Score or Sexual Health Inventory for Men questionnaire. The most recent trial involved recruiting patients to perform a sequential extension study for the focal ablation of the prostate tumor under MRI/US-guided laser irradiation (NCT04240639) [103].

## 5. Conclusions and Future Outlooks

Various approaches based on PTT have been tested in preclinical studies and have shown promising results for certain cancer treatments. In this field, a large number of nanomaterials have been developed to achieve localized hyperthermia in response to NIR light irradiation. These nanomaterials can convert photon energy into heat, which allows spatiotemporal control over the therapeutic effect. It is worth noting that the PTT ability of the nanomaterials can be optimized by adjusting external parameters, such as laser localization at the tumor site, laser operating conditions (power density and irradiation time), and accumulation of PTT agent within the tumor tissue, as well as morphological parameters such as the size and shape of the nanomaterials. Furthermore, the combination of PTT with various conventional therapies can potentially treat tumors outside the laser irradiation scope and achieve better therapeutic outcomes than PTT alone, thereby reducing the dose of PTT agents required to eradicate tumors, as well as potential side effects.

Photothermal ablation based on an external laser device has been successfully employed for tumor ablation throughout the body. However, it is difficult to effectively treat lesions near large vascular structures using laser treatment without PTT agents because of the heat-sink effect that results in heat dissipation and, thus, reduced photothermal efficiency [104]. In addition, laser light-induced thermal ablation is limited to the treatment of superficial tumors because human tissue has a strong absorption coefficient in the visible light range, and, hence, damage to non-cancerous tissues is a potential risk [105,106].

To enhance the clinical utility of PTT by improving the efficacy and selectivity of laser-induced photothermal ablation, nanomaterial-mediated PTT has emerged as an innovative approach. For example, a few clinical studies have demonstrated promising outcomes in MRI/US-guided prostate cancer ablation (NCT 04240639). Nevertheless, most nanomaterials remain in the preclinical stage and require additional biosafety characterization before clinical use, such as understanding the fate of the injected nanomaterials in the short and long term, as well as their biodegradation and toxicity. Moreover, PTT research using these nanomaterials should find ways to improve the limited selectivity of PTT agents to tumor tissue because the low tumor concentration of PTT agents requires high PTT doses to ensure their therapeutic effect. Some studies have explored nanomaterials’ functionalization with PEG and targeting ligands, such as antibodies and RGD peptides to improve the structural stability, biocompatibility, and specificity for tumor tissue [107,108,109]. Even though developing functionalized nanomaterials might delay their clinical translation, these new-generation technologies have great potential as innovative materials with improved selectivity, site-specific activation, and image-guiding therapeutic functions.

In addition to advances in the PTT agents themselves, advances in light delivery are also crucial for successful clinical translation. For example, challenges facing the clinical translation of PTTs include the limited penetration depth of laser light in biological tissues (less than 1 cm), which causes ineffective treatment for deep-tissue tumors. Thus, using NIR lasers is highly recommended for PTT because of its lower scattering and absorption by the tissues, thereby enabling deeper penetration than visible light. Therefore, current strategies involve the following new generation technologies, such as (i) the use of fiber-optic NIR lasers to reach tumors, (ii) combinational PTT with surgery on a surgical bed exposed to laser irradiation, and (iii) development of PTT agents with high extinction coefficients in the NIR II (1000–1700 nm) region, which has even deeper penetration depth and higher maximum permissible exposure than NIR I (700–1000 nm), to enable the treatment and imaging of deep-tissue tumors.

In summary, by innovating next-generation technologies, such as improved laser fiber devices (multiple interstitial fibers) and suitable PTT agents with safety, optimized optical properties, and tumor-specific targeting, better cancer treatments can be achieved. Careful consideration of combining PTT with secondary treatment is required to maximize the therapeutic effects. In this way, there is a substantial possibility of transferring new PTT platforms from the laboratory to clinics and widening their clinical application with innovative design and strategic improvements.

## Figures and Tables

**Table 1:** Representative nanomaterials used as PTT agents in combination therapies.

Nanomaterials	Combination Therapy	Mechanism	Ref.
CP-TPP/Au/PEG nanospheres	PTT + PDT	LTH (808 nm) and ROS (630 nm)	[55]
GNc-HyNA	PTT + PDT	LTH (808 nm) and ROS (690 nm)	[56]
GNS-PEG-Ce6	PTT + PDT	LTH (671 nm) and ROS (671 nm)	[57]
Te-NDs	PTT + PDT	LTH and ROS (785 nm)	[58]
UCNPs-NGO/ZnPC	PTT + PDT	LTH (808 nm) and ROS (630 nm)	[59]
CDAuNs	PTT + CT	LTH (808 nm) and CDR	[60]
DINPs	PTT + CT	LTH (808 nm) and CDR	[61]
HPSN-Pax/PdPc	PTT + CT	LTH (730 nm) and CDR	[62]
Polydopamine-rGO-MSN	PTT + CT	LTH (808 nm) and CDR	[63]
Polypyrrole@MIL-100/DOX	PTT + CT	LTH (808 nm) and CDR	[64]
HCuSNPs-CpG	PTT + IT	LTH (900 nm) and SI using small-molecule inhibitors	[65]
OVA-ICG	PTT + IT	LTH (808 nm) and SI as cancer vaccine	[66]
PCN	PTT + IT	LTH (808 nm) and SI as immune agonists	[67]
PEGylated SWNT	PTT + IT	LTH (808 nm) and SI as immune checkpoint blockades	[68]
piTRLs	PTT + IT	LTH (808 nm) and SI using immunostimulant	[69]
CSA	PTT + RT	LTH (808 nm) and X-ray	[54]
Dox-HGNP	PTT + RT	LTH (800 nm) and X-ray	[70]
mPEG@HGNPs	PTT + RT	LTH (808 nm) and X-ray	[71]
PtNP	PTT + RT	LTH (808 nm) and X-ray	[72]
WS_2_QDs	PTT + RT	LTH (808 nm) and X-ray	[73]

**Abbreviations:** CDAuNS: cancer cell membrane-coated doxorubicin-incorporated gold nanocages, CDR: chemo drug release, CpG: cytosine-guanine, CP-TPP: poly(cyclotriphosphazene-co-tetraphenylporphyrin-co-sulfonyldiphenol) nanospheres, CSA: dumbbell-shaped heterogeneous copper selenide-gold nanoparticles, CT: chemotherapy, DINP: PLGA-lecithin-PEG NPs containing DOX and ICG, DOX: doxorubicin, Dox-HGNP: doxorubi-cin-loaded hollow gold nanoparticle, GNc-HyNA: gold-nanoclustered hyaluronan nanoassembly, GNS-PEG-Ce6: chlorin e6-conjugated gold nanostars, GO-PEG-PEI-Ure B: PEG and PEI modified graphene oxide containing urease B, HCuSNPs: hollow Cus nanoparticle, HGNP: hollow gold nanoparticles, HPSN: hollow structured polymer-silica nanohybrid, ICG: indocyanine green, IT: immunotherapy, LTH: light-triggered hyperthermia, NGO: nanographene oxide, OVA: ovalbumine, Pax/PdPc: paclitaxel and palla-dium phthalocyanine, PCN: CpG-integrated OVA@Au nanorod, PEG: polyethylene glycol, PEG-[64Cu]CuS NPs: Copper 64 tagged PEG-coated copper sulfide nanoparticles, piTRLs: poly I:C- and ICG containing thermal responsive liposomes, PLGA: poly(lactic-co-glycolic acid), PtNP: platinum nanoparticles, PTT: photothermal therapy, RT: radiotherapy, ROS: reactive oxygen species generation, SI: stimulation of immune system, SWNT: single-walled carbon nanotubes, Te-NDs: tellurium nanodots, TNP-1: copper-palladium alloy tetrapod nanoparticles, UCNPs: upconversion nanoparticles, WS2QDs: tungsten sulfide quantum dots, X-ray: X-ray irradiation, ZnPc: zinc phthalocyanine.

**Table 2:** A clinical trial of nanomaterial-mediated PTT for cancer therapy.

Name (Company)	Particle Type	Indication	Clinical State on Clinical Trial.Gov Identifier
AuroLase ^®^ (Nanospectra Biosciences)	PEG-coated silica-gold nanoshells (AuroShell ^®^) for NIR-facilitated thermal ablation	Solid primary and/or metastatic lung tumors	2016 (NCT01679470- terminated)
		Refractory and/or recurrent tumors of the head and neck	2017 (NCT00848042- completed)
		Neoplasms of the prostate	2019 (NCT02680535- active, not recruiting) 2020 (NCT04240639- recruiting)

## Data Availability

Data sharing not applicable.
